# Genomic linkage map of the human blood fluke *Schistosoma mansoni*

**DOI:** 10.1186/gb-2009-10-6-r71

**Published:** 2009-06-30

**Authors:** Charles D Criscione, Claudia LL Valentim, Hirohisa Hirai, Philip T LoVerde, Timothy JC Anderson

**Affiliations:** 1 Department of Biology, Texas A&M University, College Station, TX 77843, USA; 2 Departments of Biochemistry and Pathology, University of Texas Health Science Center, San Antonio, Texas 78229, USA; 3 Department of Genetics, Southwest Foundation for Biomedical Research, San Antonio, Texas, 78245, USA; 4 Primate Research Institute, Kyoto University, Inuyama, Aichi 484-8506, Japan

## Abstract

**Background:**

*Schistosoma mansoni *is a blood fluke that infects approximately 90 million people. The complete life cycle of this parasite can be maintained in the laboratory, making this one of the few experimentally tractable human helminth infections, and a rich literature reveals heritable variation in important biomedical traits such as virulence, host-specificity, transmission and drug resistance. However, there is a current lack of tools needed to study *S. mansoni*'s molecular, quantitative, and population genetics. Our goal was to construct a genetic linkage map for *S. mansoni*, and thus provide a new resource that will help stimulate research on this neglected pathogen.

**Results:**

We genotyped grandparents, parents and 88 progeny to construct a 5.6 cM linkage map containing 243 microsatellites positioned on 203 of the largest scaffolds in the genome sequence. The map allows 70% of the estimated 300 Mb genome to be ordered on chromosomes, and highlights where scaffolds have been incorrectly assembled. The markers fall into eight main linkage groups, consistent with seven pairs of autosomes and one pair of sex chromosomes, and we were able to anchor linkage groups to chromosomes using fluorescent *in situ *hybridization. The genome measures 1,228.6 cM. Marker segregation reveals higher female recombination, confirms ZW inheritance patterns, and identifies recombination hotspots and regions of segregation distortion.

**Conclusions:**

The genetic linkage map presented here is the first for *S. mansoni *and the first for a species in the phylum Platyhelminthes. The map provides the critical tool necessary for quantitative genetic analysis, aids genome assembly, and furnishes a framework for comparative flatworm genomics and field-based molecular epidemiological studies.

## Abstract 2

The first genetic linkage map of Schistosoma mansoni reveals insights into higher female recombination, confirms ZW inheritance patterns and recombination hotspots.

## Background

**Figure 1:**
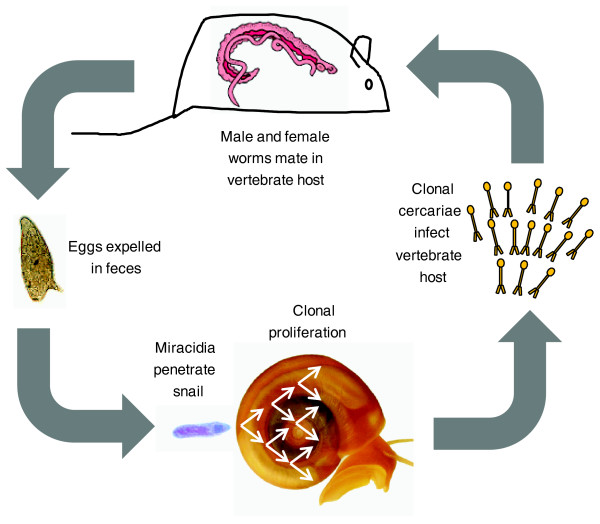
*Schistosoma mansoni *life cycle. The life cycle involves both an aquatic snail intermediate (*Biomphalaria *spp.) and a human definitive host. Mice and hamsters can be used to maintain the life cycle in the laboratory. Male (broad pink and red) and female (skinny pink) adult worms are found in the venules draining the intestine. Eggs pass through the intestine and out of the body with the feces. The eggs hatch in fresh water, and motile miracidia actively search for snails. Following penetration into the snail host, miracidia differentiate into sporocysts. Sporocysts proliferate asexually in the snail, eventually releasing motile clonal cercariae into the water. Cercariae penetrate the unbroken skin of a mammalian host, and then migrate through the bloodstream to the hepatic portal system where they develop into adults. In the laboratory, the entire life cycle takes 75 to 90 days to complete. *S. mansoni *is a conventional dioecious diploid, except for the fact that larval forms replicate asexually within the snail intermediate host. This aids in the staging of genetic crosses because clonally generated male and female larvae from different snails can be used to infect mice.

New research tools are urgently needed to combat the neglected global disease of schistosomiasis [1,2], which is caused by blood flukes in the genus *Schistosoma*. Over 200 million people across Africa, Asia, and South America are infected and recent reevaluation of disability-adjusted life year estimates indicates that schistosomes are a major global burden [1]. *Schistosoma mansoni *is one of the four major species of medical importance and infects over 83 million people in Africa and the Middle East [3]. It is the only human schistosome that has invaded the New World, with endemic transmission established in the Caribbean and Brazil, where over 6 million are estimated to be infected [4,5]. The complete life cycle of this parasite can be maintained in the laboratory using snail (*Biomphalaria glabrata*) and rodent hosts (Figure 1), thus making it one of the few experimentally tractable human helminth infections. Despite its medical importance and experimental tractability, research funding for this parasite lags far behind other tropical parasite diseases such as malaria. A well developed genetic toolkit for this parasite will help stimulate much needed research on *S. mansoni*.

Linkage mapping has been very successful for mapping the genes underlying phenotypic variation in a number of parasitic organisms. In malaria parasites (*Plasmodium falciparum*) three genetic crosses have now been completed, and a detailed microsatellite based map generated. The linkage map has resulted in the identification of major genes underlying resistance to chloroquine, quinine, sulfadoxine, host specificity, and male gametocytogenesis [6]. Similarly, linkage maps of the parasitic protozoans *Toxoplasma *[7] and *Eimeria *[8] have resulted in mapping of quantitative trait loci underlying acute virulence, while trypanosome linkage maps have also been created [9,10]. Linkage maps have been developed for a number of plant parasitic nematodes [11,12]. However, to date there are no genetic linkage maps for a helminth parasite of humans, or platyhelminths of any species.

We describe a genetic linkage map for *S. mansoni*, which we constructed for the following reasons. First, a map will aid in the assembly of the genome sequence. The present version (version 3.1) of the genome assembly contains 19,022 scaffolds, in part due to a highly repetitive genome (45%) that inhibits further assembly [13]. Importantly, the largest 280 scaffolds comprise more than 70% of the 381 Mb in version 3.1 of the genome assembly; by placing markers in these scaffolds the majority of the genome sequence can be ordered on linkage groups by examining their segregation patterns. Second, a linkage map is the critical tool needed for quantitative trait mapping [14]. There is a rich experimental literature demonstrating heritable variation in a wide variety of biomedically important traits of *S. mansoni*, such as host specificity [15] and virulence [16], and revealing co-evolutionary interactions with the snail host [17]. Infections showing reduced cure rates following treatment with the first line drug praziquantel have been observed from multiple foci, and worms recovered from these infections show increased tolerance to praziquantel in the laboratory, leading to worries about the potential for spread of drug resistance [18]. Furthermore, resistance to oxamniquine has been selected in natural parasite populations [19]. We note that this parasite is particularly well suited to linkage mapping approaches because large numbers of progeny can be recovered from single crosses, allowing statistically powerful experimental designs. In addition, clonal amplification of larvae within the snail intermediate host generates hundreds of genetically identical individuals of each recombinant genotype, allowing for precise replicated measurement of phenotypes (Figure 1). Third, with the genomes of the Asian schistosome *Schistosoma japonicum *and the free living flatworm *Schmidtea mediterranea *in the pipeline [13,20], comparative linkage mapping and synteny analysis among platyhelminths will be feasible. Given the medical and veterinary importance of many flatworm species and the diversity of life styles (parasitic, free-living, monoecious, dioecious, clonal propagation, regeneration), comparative flatworm genomics will provide a fundamental framework for tackling both applied and basic questions. Finally, the development of molecular markers spanning the genome will enable more accurate estimates of population genetic and recombination parameters from field collected parasites. In turn, a better understanding of parasite transmission among human or reservoir hosts will be gained from field-based molecular epidemiological studies of *S. mansoni *[21].

## Results and discussion

We developed a genetic map by crossing a female *S. mansoni *from the NMRI (Puerto Rico) line to a male *S. mansoni *from the LE (Brazil) line (that is, P1 grandparents). Subsequently, 2 F1 parents were crossed to generate 88 F2 progeny (41 males and 47 females). We initially designed 376 primer pairs (microsatellite loci) with at least 1 marker in the largest 283 scaffolds. Additional markers were placed in 73 of the largest 94 scaffolds to verify contig assembly and to obtain direct estimates of the recombination rate (physical distance/map distance). Screening of the grandparents and F1 parents with all 376 loci revealed that 251 loci (Additional data file 1) could be scored reliably and were informative in male and/or female meioses. All 92 individuals (88 progeny, 2 F1 parents, and 2 P1 grandparents) were genotyped with these 251 microsatellite markers. The data set was of good quality, with only 324 missing genotypes out of 22,088 possible (88 offspring × 251 loci). Each locus had an average of 86.7 offspring scored (range 80 to 88), while for each offspring an average of 247.3 loci were scored (range 221 to 251). We used the regression mapping algorithm and Kosambi mapping function implemented in JoinMap version 4 to construct the linkage map [22].

### Anchoring linkage groups to chromosomes

**Figure 2:**
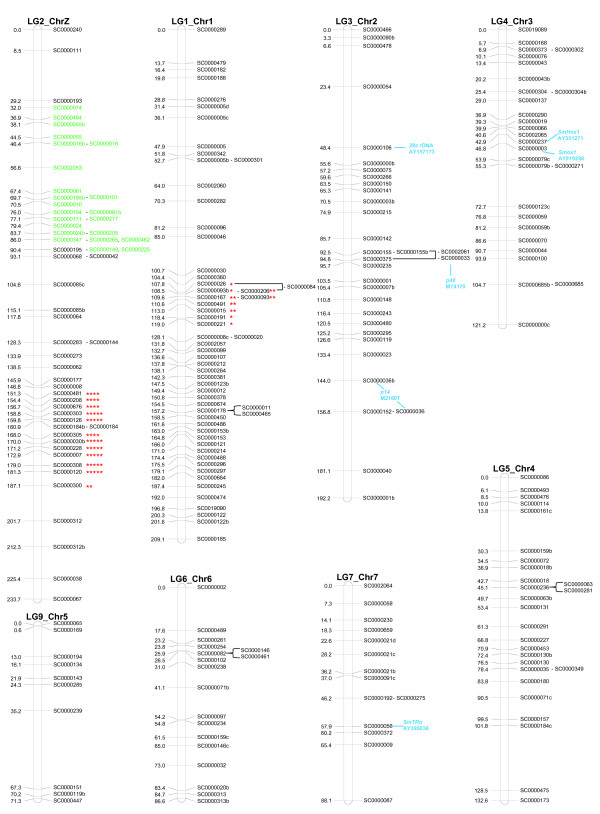
Linkage map *of S. mansoni *based on 210 markers. The map shows all 243 markers assigned to 210 unique positions on the 8 linkage groups; the numbers are map distances in centimorgans. Loci that had 0% recombination with other markers are shown adjacent to the marker used in the construction of the map. For example, marker sc84 on LG1_Chr1 had 0% recombination with both markers sc26 and sc93b. The Z-specific markers on LG2_ChrZ are shown in green. Asterisks (* *P *< 0.01, ** *P *< 0.005, *** *P *< 0.001, **** *P *< 0.0005, *****P < 0.0001) indicate significance for deviation from Mendelian expectations. Genes with previously known physical positions from fluorescent *in situ *hybridization are shown in blue with GenBank accession numbers. Blue lines show the scaffolds that match the DNA sequences of these genes in BLAST searches. These six genes add further support to the anchoring of the LG3, LG4, and LG7 to chromosomes 2, 3, and 7, respectively. See Figure 3 for comments on the match with sc3 and *Smox1*.

**Figure 3:**
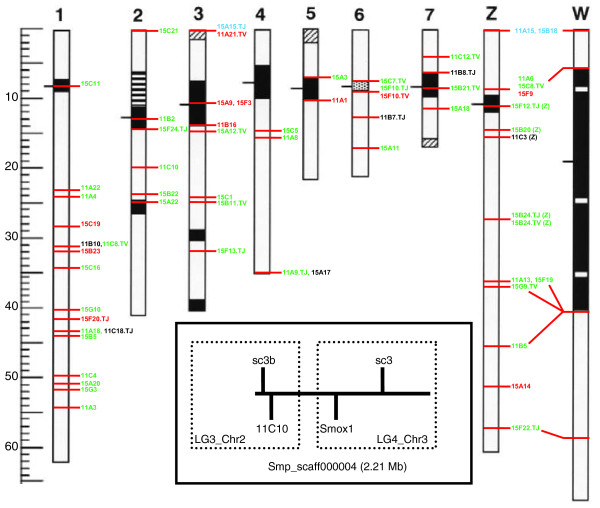
Anchoring of linkage groups to chromosomes by fluorescent *in situ *hybridization. The black and stippled regions show the heterochromatin (C-banded regions) on the seven autosomes and two sex chromosomes, the vertical lines on chromosome 2 show the rDNA, and the ruler is marked in 1 Mb increments. The chromosomal regions to which bacterial artificial chromosomes (BACs) hybridize are marked. All fluorescent *in situ *hybridization (FISH)-mapped BACs shown hybridize uniquely to a single position in the genome and BLAST match to scaffolds from which the microsatellite markers were designed (see Additional data file 4 for the BLAST matched markers for each BAC). The green BACs are congruent with linkage mapping results both in terms of chromosome and relative marker order. Hence the number of green markers provides a visual impression of the strength of support anchoring each linkage group. Black BACs are congruent with linkage mapping results for chromosome, but the ordering of markers is incongruent by a large distance (compare Figure 2 and Additional data file 4). Red BACS are incongruent (that is, the linkage mapping results and FISH identify different chromosomes). Red BACs on the same chromosome always matched to markers from different linkage groups, thus displaying a random pattern of mismatching. The blue BACs 15A15.TJ and 11A15, 15B18 indicate potential positions for the orphan markers sc117 and LG8 that were not incorporated into the linkage groups. BACs followed by TJ or TV indicate that only 1 BAC end matched correctly. TJ and TV refer to the two different BAC ends that could be sequenced and follow the naming convention given in GenBank. The BACs followed by a (Z) on chromosome Z indicate BACs that match to Z-specific scaffolds. The assignment of LG9 to chromosome 5 is tentative as there was only one congruent and one incongruent marker. The inset figure illustrates 1 of 16 scaffolds where markers on the same scaffold mapped to different linkage groups. The schematic of Smp_scaff000004 (2.21 Mb) shows the relative positions of two FISH markers (BAC 11C10 and *Smox1*) and two linkage markers (sc3 and sc3b). Both sets of markers suggest that this scaffold was incorrectly assembled (see Figure 2 for the FISH result of *Smox1*).

In a sex-combined map, 243 of 251 markers (97%) assembled into 8 major linkage groups of 10 or more markers (Table 1, Figure 2). The remaining 8 of the 251 markers did not fall into these 8 linkage groups: 5 clustered in 2 small linkage groups (of 2 and 3 loci) while the remaining 3 markers were unlinked (Additional data file 2). The *S. mansoni *genome (300 Mb) consists of 7 pairs of autosomal chromosomes and 1 pair of sex chromosomes (female = ZW, male = ZZ). ZW refers to systems in which the female is the heterogametic sex as opposed to XY in which males are the heterogametic sex. In conjunction with fluorescence *in situ *hybridization (FISH) data of bacterial artificial chromosomes (BACs) or known genes, we could anchor seven of the eight major linkage groups to chromosomes with high confidence (Figures 2 and 3). Linkage group 9 (LG9) was tentatively called chromosome 5 by elimination. However, the lack of FISH markers on that chromosome prevents definitive assignment of a linkage group to chromosome 5. In some instances, we found that mapped markers and FISH-mapped BACs were not congruent (red BACs in Figure 2). This incongruence could be due to inaccurate FISH hybridization, mislabeling of BAC clones, or incorrect genome contig assembly (discussed below). However, our data do not permit the identification of the causative factor(s). Ordering of loci within these eight chromosomal linkage groups was conducted after retaining a single marker from sets of loci showing identical segregation patterns (that is, 0% recombination; Table 1). The mean chi-square values, a measure of the goodness-of-fit of the regression mapping to the pairwise estimates of recombination frequencies, were well below 1 (range 0.105 to 0.341) for all the linkage groups. This indicates that there was good support for the ordering of markers within each linkage group (Additional data file 2).

**Table 1:** Summary of linkage groups

LG_Chr*	Chromosome size (Mb)^†^	Total markers^‡^	Mapped markers^§^	Map length (cM)	Adjusted map length (cM)	Interval spacing (cM/interval)^¶^
LG1_Chr1	62.4	58	51	209.15	218.95	4.18
LG3_Chr2	41.2	32	28	192.24	204.98	7.12
LG4_Chr3	40.8	28	24	121.16	132.04	5.27
LG5_Chr4	35.1	27	24	132.65	144.03	5.77
LG9_Chr5	21.7	10	10	71.30	84.84	7.927
LG6_Chr6	21.2	18	16	86.61	98.00	5.777
LG7_Chr7	16.9	14	13	88.08	101.04	7.34
LG2_ChrZ	60.7	56	44	233.65	244.70	5.437
						
Total	300	243	210	1134.84	1228.59	5.62

*LG, linkage group; Chr, probable chromosome based on fluorescent *in situ *hybridization data. ^†^Physical chromosome size was based on the relative size of chromosomes [48] and an estimated 300 Mb genome size. ^‡^Total number of markers in each linkage group. ^§^When two or more markers had 0% recombination, we selected a single marker to generate the maps. ^¶^Calculated as the unadjusted map length divided by the number of intervals (mapped markers minus 1). The interval spacing reported under the total is 1,134.84 cM divided by 202 intervals.

### Recombination parameters and map length

The final genetic map of the 8 major linkage groups (Table 1) contained 210 loci because 33 loci showed identical segregation to other loci (Figure 2). The 8 chromosomal linkage groups spanned 1,134.8 cM with an average marker spacing of 5.6 cM per interval. To account for linkage group ends beyond terminal makers, we used the methods in [23,24], which calculate an expected map length for the terminal regions of the linkage groups (see Materials and methods). These adjustments yielded a total adjusted genome length of 1,228.6 cM (Table 1). Linkage groups ranged in (adjusted) size from 84 to 244 cM. The expected distance of a gene, *E(m)*, from the nearest random marker (n = 210) is 2.9 cM with an upper 95% confidence interval (CI) of 8.7 cM [14].

**Figure 4:**
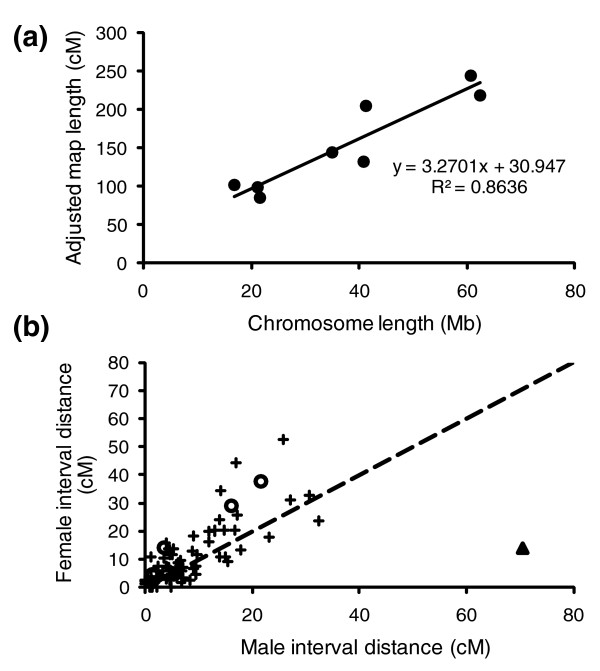
Recombination rates in *S. mansoni*. **(a) **Relationship between adjusted map length and physical size of chromosomes. The positive relationship (*P *= 0.0008) indicates that the average recombination rates are comparable among chromosomes. **(b) **Comparison of female and male recombination rates. Plus signs indicate comparisons among 78 autosomal intervals. The recombination rate is 1.27-fold higher in females than in males (*P *= 0.019) for these intervals. For comparison, the open circles are the four pseudoautosomal intervals on the sex chromosomes and the triangle is the interval over the Z-specific region.

There was a strong positive relationship (*r*^2 ^= 0.86, *P *= 0.0008) between the physical size (determined by cytology; Table 1) and genetic map lengths of the chromosomes (Figure 4a), indicating that the average recombination rates are comparable among chromosomes. We made two estimates of recombination rate with our data set. The first, 244.2 kb/cM, is the physical genome size divided by adjusted map length. The second is based on 24 mapped distance intervals between markers that were placed on the same scaffolds of version 3.1 of the genome assembly (Additional data file 3). This provided a direct estimate of physical distance to map distance of 227.2 kb/cM (95% CI 181 to 309, based on 10,000 Monte Carlo replicates of intervals). These estimates are the first for a representative of the phylum Platyhelminthes and indicate that recombination per physical distance in *S. mansoni *is comparable to other multicellular invertebrates of similar genome size [25]. Interestingly, the negative relationship between recombination rate and physical genome size given in [25] predicts a very similar rate of 302 kb/cM for *S. mansoni*. Our estimates are also consistent with recombination frequencies obtained from previous cytogenetic work [26]. The average chiasma frequency of *S. mansoni *was estimated at 18.3 (95% CI 17.3 to 19.3) [26], which equates to total map lengths from 865 to 965 cM and recombination rates from 346.8 to 310.9 kb/cM. Thus, the cytogenetic estimate is marginally lower than our genetic estimate. In part, the cytogenetic estimates of recombination may be biased downward as chiasma frequencies were only measured in males, in which recombination is reduced (see below).

We were also able to compare 78 autosomal intervals between male and female meioses (Figure 4b) to obtain sex-specific recombination rates. Over these homologous regions, the average female interval (9.42 cM) was significantly longer than the average male interval (7.42 cM) (*P *= 0.019, Wilcoxon signed-rank test). Sex-biased recombination rates (heterochiasmy) have been reported in many organisms (reviewed in [27,28]). The evolutionary hypotheses and mechanistic processes put forth to explain sex differences in recombination can be difficult to disentangle [28]. However, as the female, the heterogametic sex, had 1.27-fold higher recombination than the male, we can rule out the Haldane-Huxley rule. This rule predicts lower recombination among autosomes in the heterogametic sex because selection acts against recombination between different sex chromosomes. Our data provide a second and phylogenetically independent example of a ZW system that is inconsistent with the Haldane-Huxley rule (the other is in the passerine bird *Acrocephalus arundinaceus *[29]).

### Genome assembly by linkage

Of the 243 markers assembled on the 8 chromosomes, there are 203 unique scaffolds (totaling 209 Mb) represented. Thus, the linkage map contains 70% of the estimated 300 Mb physical genome and 55% of the 381 Mb currently in version 3.1 of the genome assembly. However, the current genome assembly contains considerable redundancy and overestimates genome size and the 55% is thus likely to underestimate true coverage. Furthermore, if the total genetic map length is calculated from the direct estimate of the recombination rate (300 Mb/227.2 kb/cM = 1,320 cM), then the unadjusted map length accounts for 86% (1,134 of 1,320 cM) of the total genetic map length. The genome assembly will benefit from the broad coverage of the map, high density of markers, and placement of previously unanchored and unordered scaffolds. The map data also provide a means to assess the quality of the current assembly. There were 37 scaffolds with 2 or more markers located < 2.2 Mb apart (Additional data file 3), which equates to about 8 to 10 cM. Markers from 21 of the scaffolds were consistent with this pattern. However, there were 16 scaffolds where markers mapped to different linkage groups or had map distances that were much greater than expected based on the recombination rate (Additional data file 3; Figure 2). These data suggest that a substantial portion (43% of our sample) of the current assembly is incorrect. However, given the highly repetitive nature of the genome, it is encouraging that 57% of the scaffolds were valid and that many of the mapped markers show congruence with FISH-mapped BACs (Figure 3). These results also illustrate the utility of linkage maps in correcting genome assembly errors. Thus, the map will provide a platform for the continued assembly for the genome.

### Marker segregation on sex chromosomes

**Figure 5:**
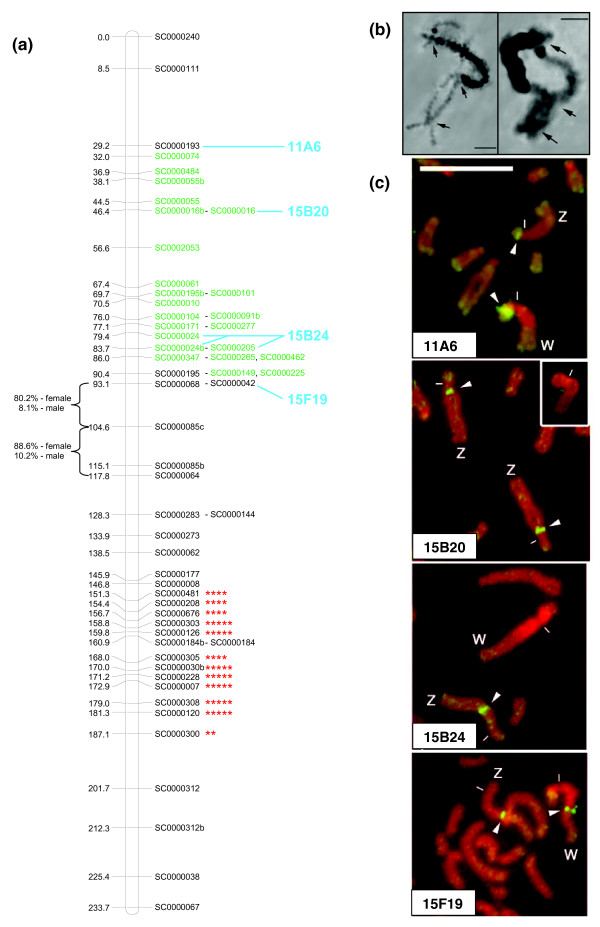
Z chromosome features. **(a) **Map of the Z chromosome. Loci that had 0% recombination with other markers are shown adjacent to the marker used in the construction of the map. The Z-specific markers are shown in green. Asterisks (*P < 0.01, **P < 0.005, ***P < 0.001, ****P < 0.0005, *****P < 0.0001) indicate significance of deviation from Mendelian expectations, where brackets show recombination hotspots in the female meioses (recombination frequencies for each sex are listed next to the brackets). **(b) **Meiotic metaphase spreads from females showing the Z and W bivalents. This figure illustrates the non-recombining region between the Z and W chromosomes. The dark staining regions are heterochromatin of the W chromosome and the large black arrows mark chiasmata. Scale bars are 10 μm. **(c) **Fluorescent *in situ *hybridization (FISH) showing the hybridization position of bacterial artificial chromosome (BACs; names at lower left of each panel) that BLAST to scaffolds with mapped microsatellite markers. The white arrowheads show BAC hybridization and the white dash is the centromere. Scale bar is 10 μm. The inset for BAC 15B20 is the W chromosome, on which 15B20 does not hybridize (that is, it is Z-specific). The genetic map position of the markers on these BACs is shown in blue text in (a). FISH allows assignment of linkage groups to physical chromosomes (see also Additional data file 4 and Figure 3).

There are several interesting features on LG2 of the Z chromosome (LG2_ChrZ; Figure 5a). Previous cytogenetic data suggested that the heterochromatin region of the W chromosome does not recombine with a region on the Z chromosome, but that there are two flanking pseudoautosomal regions (Figure 5b). This was confirmed in our linkage map by the identification of 23 Z-specific markers on 20 unique scaffolds that clustered in a group (green markers in Figure 5a) and were flanked by pseudoautosomal regions on either side. All female worms that were genotyped had a single allele and the alleles present in the F1 female parent and F2 female progeny were always inherited from their respective male parent. In contrast, male worms could be heterozygous. These patterns are consistent with females being hemizygous at these loci. FISH mapping confirms the close proximity of the pseudoautosomal markers sc68, sc42, and sc193 at the borders of the heterochromatin region on the W (Figures 3 and 5c; Additional data file 4). Furthermore, the male meioses showed extensive recombination across the Z-specific region in comparison to the female meioses (triangle in Figure 4b). In contrast, the female recombination was greater in pseudoautosomal regions that bordered the Z-specific region (sc240-sc111, sc111-sc193, sc195-sc68, sc68-sc64; shown as circles in Figure 4b). This latter pattern is consistent with the higher female autosomal recombination rate. It is plausible that the higher female recombination rates in the pseudoautosomal regions that border the Z-specific region may be a mechanistic consequence of limited areas for chiasma formation between the Z and W chromosomes in female meioses. Consistent with this idea, we observed potential hot spots of recombination on either side of sc85c that occur in female but not male meioses. Estimated recombination frequencies between sc68 and sc85c, and sc85c and sc64 were 8 and 10% in the male, respectively. In the female, they were 80 and 88% (Figure 5a.). Further support for these recombination hot spots comes from the presence of 18 double recombinant genotypes (from F1 female gametes) that involved sc85c, and 120 pairwise comparisons that show excess recombination (> 60%) between markers in the region from sc208 to sc312 to markers in the region of sc195 to sc240 (Additional data file 2).

### Segregation distortion

Two regions in the linkage map showed strong deviations from Mendelian inheritance (χ^2^-test, α = 0.01): 12 markers between sc300 and sc481 on LG2_ChrZ, and 9 markers between sc221 and sc26 on LG1_Chr1 (Figures 2 and 5a). The remaining 222 markers did not deviate from Mendelian expectations. The 2 regions displayed different patterns of distortion. From sc481 to sc126 on LG2_ChrZ (Figure 2), there was an excess of heterozygous genotypes of an allele from the NMRI female and LE male. From sc305 to sc120, however, there was a major decrease in the NMRI female homozygote genotype (only one to six individuals). The pattern on LG1_Chr1 was uniform across loci in having a decreased NMRI female homozygote genotype and one heterozygote combination, whereas the other heterozygote combination was normal and the LE male homozygote was increased (Figure 2). It is not uncommon to find genomic regions with segregation distortion when crossing diverged populations due to the evolution of coevolved gene complexes or of incompatible regions [24,30,31]. The NMRI and LE lines have been separated well over 250 generations in the laboratory (see Materials and methods). Genetic load from inbreeding depression that may build up in laboratory maintained lines is another plausible explanation [32]. If loci between the two regions were interacting (for example, an allele is deleterious at one locus only in the presence of a particular allele at another locus), we would expect genotypic associations between markers in the two regions. However, pairwise comparisons failed to detect any genotypic associations (*P *> 0.14 in all comparisons). Thus, the cause of distortion at the two regions appears to be independent.

## Conclusions

The linkage map complements the genome sequence and other tools such as RNA interference, and adds to a growing toolkit for genomic analyses in *S. mansoni*. We anticipate that next generation sequencing and rapid single nucleotide polymorphism typing methods will be used to build on the foundation provided by this microsatellite-based map. In particular, next generation sequencing of single parasite genotypes (rather than pooled individuals from laboratory parasite lines) will allow rapid improvements in the genome assembly that can be verified by genotyping single nucleotide polymorphisms in the genetic cross. The combination of these tools will improve the genome assembly and provide markers for fine mapping of genes that underlie traits of biological or biomedical interest. We also foresee that provision of these tools will invigorate research on this pathogen and attract researchers from other fields. A great advantage to studying *S. mansoni *over other human helminths is that the complete life cycle can be maintained in the laboratory using mice or hamsters as the definitive host, thus allowing experimental investigation of life cycle traits (for example, [15,16,33]). Such studies have demonstrated that numerous phenotypic traits of *S. mansoni *vary within and between parasite populations and that many of these traits have a genetic basis. Linkage mapping, utilizing the 5 cM map described here, provides a means to investigate the underlying basis of traits of medical and epidemiological relevance, such as virulence, host specificity, and drug resistance. For example, different strains of *B. glabrata *and *S. mansoni *have been shown to have different compatibilities in terms of infectivity or virulence [15,17]. Drug resistance is a trait of particular biomedical interest and this trait can readily be measured both *in vivo *in infected rodents and *in vitro *using adult worms maintained in culture media. Resistance to oxamniquine has been demonstrated as a double recessive trait in *S. mansoni *[33], and there is clear evidence that parasites with increased tolerance to the first-line drug praziquantel occur in natural populations [18]. Linkage mapping will allow identification of the genes responsible for resistance to these drugs. For mapping the genes underlying these traits additional crosses will need to be conducted. The microsatellite markers used for map construction are highly variable, so the majority of markers are likely to be informative in additional crosses. The map also has multiple applications for developmental and evolutionary biology. Provision of hundreds of molecular markers and recombination parameters will facilitate high resolution population genetic studies of *S. mansoni*, which will improve our understanding of transmission patterns in endemic areas. The *S. mansoni *linkage map presented expands the genetic toolkit for *S. mansoni*, providing opportunities to understand fundamental features of *S. mansoni *biology, and opening doors to new advances in combating this human pathogen.

## Materials and methods

### Genetic cross

We crossed a NMRI female to an LE male to generate F1 progeny. Subsequently, a male and female from the F1 were crossed to generate 88 F2 progeny (reared to the adult stage). The NMRI line originated in the early 1940s from human isolates in Puerto Rico and the LE line was established from a human isolate in 1965 in Belo Horizonte, Brazil [34]. At each stage in the cross, we conducted monomiracidial infections of snails (*B. glabrata*). Because sex is determined in the zygote (which develops into a miracidium) by a chromosomal mechanism, monomiracidial infections allowed us to be certain that we were using single clonal types (that is, single genetic individuals of the same sex) in the crosses. After 28 days (the last 3 under darkness), snails were exposed to light to shed cercariae. Cercariae were sexed with the following protocol. We collected 20 to 50 cercariae of one clonal genotype from each infected snail. For DNA extractions, samples were placed in 50 μl of 5% chelex containing 0.2 mg/ml of proteinase K, incubated for 2 h at 56°C, and boiled at 100°C for 8 minutes. PCR with the W1 primers [35], which are specific to a repetitive region on the W chromosome in females, was used to discriminate between males and females. PCR was performed with 15 μl reactions containing 2.4 μl of extraction supernatant, 1× PCR buffer, 1.5 mM MgCl_2_, 0.2 mM of each dNTP, 0.4 μM of each primer, and 0.75 units (0.15 μl) Taq DNA polymerase (Takara Shuzo Co., Otsu, Shiga, Japan). PCR cycling was 95°C for 3 minutes, once; 94°C for 45 s, 54°C for 30 s, 72°C for 45 s, 35 times; 72°C for 7 minutes, once. Because this test depends on the failed amplification in males, we ran a concurrent PCR under the same conditions with the autosomal locus sc18 (see Additional data file 1 for primers) to ensure that the DNA had successfully been extracted from each sample. Results were visualized on a 2% agarose gel containing GelStar^® ^nucleic acid gel stain (Lonza, Basel, Switzerland).

Upon identification of gender, snails were shed again to collect cercariae for infections. We exposed a hamster to 300 female cercariae (one genetic individual) and 300 male cercariae (one genetic individual) for the parental cross. After 45 days, the hamster was euthanized and perfused to collect adult worms. Eggs were collected from the liver and hatched under light to obtain miracidia for the next generation of monomiracidial snail infections. This process was repeated to stage the F1 cross. In the F2 generation we reared worms to the adult stage in mice (BALB/c). Mice were exposed to 200 female cercariae (one genetic individual) and 200 male cercariae (one genetic individual) or with 200 cercariae of a single sex. F2 worms were collected from mice after 40 days.

### Genomic DNA extraction and whole genome amplification

Individual adult worms were placed in 50 μl of 5% chelex containing 0.2 mg/ml of proteinase K, incubated for 2 h at 56°C, and boiled at 100°C for 8 minutes. The GenomiPhi V2 DNA amplification kit (GE Healthcare, Piscataway, New Jersey, USA) was used to amplify whole genomic DNA according to the manufacture's protocol.

### Microsatellite markers and genotyping

Microsatellite markers were designed from the largest 283 supercontigs in version 3.0 of the genome assembly. These 283 supercontigs account for 72% of sequence data in version 3.1 of the genome assembly (available from the Sanger Institute [36]). The difference in the two versions is only the removal of approximately 720 kb of sequence in version 3.1, most of which (703 kb) was a single supercontig that was removed. The major change was the renaming of supercontigs to scaffolds without change to the actual sequence data. We provide this information in Additional data file 1. Markers were selected from a masked copy of the genome to avoid placing markers in repetitive DNA. Tandem Repeats Finder version 4 [37] was used to search the contigs for microsatellite repeats. Only perfect di- and trinucleotide repeats were selected. Primer 3.0 [38] was used to design all primers with an annealing temperature of 54 to 56°C.

We used the M13(-21) method for genotyping [39]. The M13(-21) oligonucleotide was added to the 5' end of each forward primer. We also 'pig-tailed' the reverse primers by adding GTTTCTT to the 5' ends [40]. PCR was performed in 5 μl reactions containing 15 ng of genome amplified template, 1× PCR buffer, 1.5 mM MgCl_2_, 0.2 mM of each dNTP, 0.08 μM of the forward primer, 0.16 μM of the reverse primer, 0.16 μM of the fluorescent labeled M13(-21) primer, and 0.15 units (0.03 μl) Taq DNA polymerase. PCR cycling was 94°C for 5 minutes, once; 94°C for 30 s, 56°C for 45 s, 65°C for 45 s, 30 times; 94°C for 30 s, 53°C for 45 s, 65°C for 45 s, 8 times; 65°C for 10 minutes, once. PCR products were run on an ABI 3100 with Genescan software and scored using Genotyper (Applied Biosystems, Foster City, CA, USA). LIZ500 size standard (Applied Biosystems) was used for all loci. All traces were visually examined and checked for correct peak labeling.

All loci are named for the supercontig (version 3.0 of the genome assembly) on which they reside. Additional data file 1 provides the cross reference information to the scaffold (version 3.1 of the genome assembly) that the markers are on. In addition, Additional data file 1 provides the information on repeat motifs, primer sequences, linkage map positions, scaffold (version 3.1) length, physical position of the microsatellite repeat motif on the scaffold (version 3.1), and physical positions of the flanking sequences used to design primers. Marker names with lower case letters indicate that more than one marker was placed on that supercontig. This lettering does not indicate physical ordering of markers on supercontigs. For example, sc5, sc5b, sc5c, and sc5d are all markers on Supercontig_0000005 (Smp_scaff000005), but not necessarily in that physical order. For simplicity, we abbreviate marker names in the text (for example, sc5b); however, the names are written in full in the figure maps and tables to facilitate queries that match the genome database. A list of the 37 scaffolds that have more than one mapped marker is in Additional data file 3.

### Linkage map construction

We used JoinMap [22] both to assign markers to linkage groups and then to order markers on each linkage group. The F1 parents and F2 offspring were coded according to the CP population type, a population resulting from a cross between two heterogeneously heterozygous and homozygous diploid parents. We input the phase of the F1 genotypes based on the genotypes of the grandparents. Z-specific markers, which were identified by the fact that all females were hemizygous with an allele inherited from their male parent, were coded as nnxnp (F1 female × F1 male). We generated a sex-combined map irrespective of whether the locus was informative in one or both of the F1 parents.

#### Assignment and ordering of markers to linkage groups

Overall, there was strong support for each linkage group and the ordering of markers within each linkage group (Additional data file 2). Linkage groups were formed at a threshold pairwise recombination frequency of 30%. This threshold corresponded to an independence LOD (a description of this calculation is given in [22]) of 4 or greater for each linkage group except for LG5_Chr4 (Additional data file 2). LG5_Chr4 had 25 markers that were grouped at an independence LOD of 10 but markers sc475 and sc173 were not among them. However, these 2 markers were within the 25% threshold of the pairwise recombination frequency. Visual inspection of the estimated recombination frequencies and FISH data supported the inclusion of sc475 and sc173 in LG5_Chr4 (Figure 2).

Prior to ordering markers within linkage groups, we identified loci that had 0% recombination with one or more markers. In such cases, we retained only one marker for subsequent analyses (Additional data file 2), choosing the locus that was more informative and/or had fewer missing genotypes. We used the Kosambi mapping function to convert recombination frequencies into map distances. The regression mapping algorithm with the default settings (recombination frequency threshold < 0.4, LOD threshold > 1) was used to order loci within each linkage group. On LG3_Chr2, a reduced stringency (recombination frequency threshold < 0.49, LOD threshold > 0.1) was needed to include markers sc54 to sc466 (Additional data file 2). Visual inspection of the estimated recombination frequencies and FISH data supported the order of these markers. A ripple (all ordering permutations within a moving window of three adjacent markers) was performed after the addition of each new marker. When the best position of a marker decreased the goodness-of-fit too sharply (default jump = 5) or gave rise to negative distance estimates, the locus was removed. After all loci are handled once, a second round is made to add previously excluded loci using the added information of all pairwise markers included in the first round. In a third round, all loci previously removed are added to the map without constraints in order to obtain a general idea about where poorer fitting loci reside on the map. All linkage groups except LG2_ChrZ had a single round of mapping. Marker sc193 was the only marker in LG2_ChrZ that needed a second and third round. However, FISH data and visual inspection of the estimated recombination frequencies confirmed the relative position of this marker (Figure 5c). The overall map order can be evaluated by a goodness-of-fit measure between the direct pairwise estimates of recombination frequency and the frequencies obtained from the map (using the mapping function). This goodness-of-fit measure is roughly distributed as chi-square [22]. Mean chi-square values (Chi-square test statistic divided by the degrees of freedom) well below 1 indicate good support for the ordering of markers [22].

#### Evaluation of double recombinants and mutations

With the exception of LG2_ChrZ, there were few improbable genotypes and suspect linkages (Additional data file 2). The genotype probabilities are calculated conditional on the map and genotypes of neighboring loci [22]. These probabilities flag possible double recombinants or possible genotyping errors [22]. There were 59 genotypes with *P *≤ 0.01. We visually re-inspected all genotypes (n = 16) with *P *≤ 0.001 and confirmed that the genotypes were correctly scored. Although these could represent double recombinants, we cannot rule out mutation (naturally, genome amplified, or PCR induced) as a possible cause. For example, one genotype, which had the only *P *< 0.0001, showed a double recombinant from both the male and female meioses. Re-inspection of this genotype showed that a possible 2 bp mutation in one of the alleles of this offspring could create this possible pattern. Removal or 'assumed correction' of a subset of these genotypes, including the latter, had little impact on the loci ordering or on map length of each linkage group. Thus, we did not remove these possible double recombinants (< 0.27% of the genotypes in the data set) from the final analysis. On LG2_ChrZ, 18 of the 25 improbable genotypes involved marker sc85c. In the main text, we discuss how the regions flanking sc85c represent possible host spots of recombination in the female meioses. Supporting this claim, a large number of suspect linkages (> 60% recombination) occur on LG2_ChrZ between markers that lie in the region from sc208 to sc312 with markers in the region of sc195 to 240.

#### Estimation of linkage map parameters

To account for the terminal parts of the linkage groups, an adjusted map length for each linkage group was calculated by averaging the results from the methods of Fishman *et al*. [24] and Chakravarti *et al*. [23]. The Fishman *et al*. [24] method adds twice the average spacing of markers (across the entire map) to the lengths of each linkage group. Method 4 of Chakravarti *et al*. [23] expands each linkage group by (*m *+ 1)/(*m *- 1), where *m *is the number of loci mapped. Formula 14.8 in [14] was used to calculate the expected distance of a gene, *E(m)*, from the closest of n (= 210) random markers and the upper 95% confidence interval for this distance. The total adjusted map length of 1,228.59 cM was used as the estimate of *L*. Formula 14.7 in [14], which accounts for linear chromosomes, was in near agreement with formula 14.8, where 94.24% of the genome was within 8.7 cM of a marker assuming a random distribution of markers.

#### Sex specific recombination and segregation distortion

Parental meioses were examined by creating maternal and paternal population nodes in JoinMap. We only compared intervals between homologous loci that generated the same mapping order as the sex-combined map. Homologous map distances for all autosomal markers were compared with a Wilcoxon signed-rank test to test for a difference in male and female recombination rates. Segregation distortion (non-Mendelian inheritance) for all loci was tested in JoinMap (χ^2^-test, α = 0.01). To determine if there were interactions between the two distorted regions on LG1_Chr1 and LG2_ChrZ, we tested for an association of genotypes between pairs of markers from the two regions. We compared each of the seven mapped markers on LG1_Chr1 from sc221-sc26 to randomly chosen markers from the region of sc300-sc481 on LG2_ChrZ (that is, we conducted seven tests). To analyze the contingency tables of genotypes between loci, we used the program RxC [41]. RxC employs the metropolis algorithm to obtain an unbiased estimate of the exact *P*-value (that is, Fisher's exact test) for any sized contingency table. The following Markov chain parameters were used to test significance: 2,500 dememorizations, 100 batches, and 2,500 permutations per batch.

### Anchoring markers in the linkage map to chromosomes

Methods for FISH analysis are described in [42]. BAC end sequences obtained from GenBank (see Additional data file 4 for accession numbers) were used in BLAST searches of the genome database. We only used BACs that FISH mapped to a single homologous pair of chromosomes (or Z and W). Linkage groups were anchored to chromosomes by the following. We first determined if the FISH mapped BAC BLAST matched to a scaffold. If the scaffold was one in which we had a mapped microsatellite maker, we considered that marker to belong on the chromosome to which the BAC was FISH mapped. Evidence from several of these matches allowed us to anchor the linkage groups to chromosomes (Additional data file 4; Figure 3). We also BLAST matched six genes with known chromosomal locations (Figure 2): *28s *rDNA on chromosome 2, eggshell protein genes *p14 *and *p48 *on chromosome 2, *SmTRα *on chromosome 7, and *SmHox1 *and *Smox1 *on chromosome 3 [43-47].

## Abbreviations

BAC: bacterial artificial chromosome; Chr: chromosome; CI: confidence interval; FISH: fluorescent *in situ *hybridization; LG: linkage group.

## Authors' contributions

CC, TA, and PL designed the study. CC, TA, PL, and CV carried out experimental work. CC and CV did the molecular work. CC and TA did the data analysis. HH did the FISH work. CC and TA wrote the bulk of the manuscript, but with contributions from all authors. All authors read and approved the final manuscript.

## Additional data files

The following additional data are available with the online version of this paper: primer, motif, and position information for each microsatellite marker (Additional data file 1); summary methods, grouping statistics, and ordering of markers used in the construction of the linkage map (Additional data file 2); a list of supercontigs where more than one marker was placed (Additional data file 3); a list of FISH-mapped BACs that BLAST matched to scaffolds with markers in the linkage map (Additional data file 4).

## Supplementary Material

Additional data file 1 An Excel worksheet showing primer, motif, and position information for each microsatellite marker. Click here for file

Additional data file 2 A text document containing summary methods, grouping statistics, and ordering of markers used in the construction of the linkage map. Click here for file

Additional data file 3 An Excel file containing a list of supercontigs where more than one marker was placed. Click here for file

Additional data file 4 An Excel file containing the list of FISH mapped BACs that BLAST matched to scaffolds with markers in the linkage map. Click here for file
