# Supplementary material for: Genomic linkage map of the human blood fluke Schistosoma mansoni
Source: Genome Biol. 2009 Jun 30;10(6):R71. doi: 10.1186/gb-2009-10-6-r71 (PMC2718505; doi:10.1186/gb-2009-10-6-r71)
Supplement: Additional data file 2 — A text document containing summary methods, grouping statistics, and ordering of markers used in the construction of the linkage map. [file gb-2009-10-6-r71-S2.doc]

**Additional data file 2.** Summary methods, grouping statistics, and ordering of markers used in the construction of the linkage map.

| LG_Chr^a^ | Num. of markers^b^ | % linkage^c^ | Ind. LOD^d^ | Rounds of mapping | Mean Chisquare | Suspect linkages^e^ | Improbable genotypes^f^ | Comments |
| --- | --- | --- | --- | --- | --- | --- | --- | --- |
| LG1_Chr1 | 58 (51) | 20 | 8 | 1 | 0.260 | 0 | 12 (5) |  |
| LG3_Chr2 | 32 (28) | 25 | 4 | 1 | 0.126 | 1 | 4 (1) | Reduced stringency in regression mapping to fit markers sc54 to sc466 |
| LG4_Chr3 | 28 (24) | 20 | 9 | 1 | 0.273 | 0 | 4 (3) |  |
| LG5_Chr4 | 27 (24) | 25 | N/A | 1 | 0.124 | 0 | 8 (3) | Markers sc475 and sc173 were not included with the other 25 markers at a Ind. LOD of 3 |
| LG9_Chr5 | 10 (10) | 30 | 6 | 1 | 0.105 | 0 | 2 (0) |  |
| LG6_Chr6 | 18 (16) | 20 | 8 | 1 | 0.231 | 0 | 3 (1) |  |
| LG7_Chr7 | 14 (13) | 25 | 6 | 1 | 0.127 | 0 | 1 (0) |  |
| LG2_ChrZ | 56 (44) | 20 | 8 | 3 | 0.341 | 120 | 25 (3) | Most suspect linkages involve markers sc208 to sc312 with markers sc195 to 240; most improbable genotypes (*n* = 18) involve sc85c |
| LG8 | 3 (N/A) | 10 | 10 | N/A | N/A | N/A | N/A | sc34, sc485, sc154; could not be ordered due to segregation types |
| LG10 | 2 (N/A) | 10 | 10 | N/A | N/A | N/A | N/A | sc223, sc136 |
| unlinked | 3 (N/A) | N/A | N/A | N/A | N/A | N/A | N/A | sc117, sc170, sc23b |

^a^LG, Linkage group; Chr, probable chromosome based on FISH data.

^b^Total number of markers in each LG. In parentheses is the number of markers used to generate the map after excluding markers that had 0 % recombination with other markers in the LG.

^c^The largest pairwise recombination frequency that grouped all loci in that LG.

^d^The lowest Independence LOD score that grouped all loci in that LG.

^e^Suspect linkages are pairs of loci that have an estimated recombination frequency > 0.6.

^f^Genotype probabilities are calculated conditional on the map and genotypes of neighboring loci. These probabilities flag possible double recombinants (or genotyping errors). Shown is the total number of genotypes where *p* ≤ 0.01. In parentheses is the number of genotypes where *p* ≤ 0.001.
